# Supplementary material for: Local community assembly mechanisms shape soil bacterial β diversity patterns along a latitudinal gradient
Source: Nat Commun. 2020 Oct 27;11:5428. doi: 10.1038/s41467-020-19228-4 (PMC7591474; doi:10.1038/s41467-020-19228-4)
Supplement: Supplementary file 1 — Supplementary Information [file 41467_2020_19228_MOESM1_ESM.pdf]

Supplementary Information

Local community assembly mechanisms shape soil bacterial β diversity patterns along a latitudinal gradient

Xiao Zhang et al.

Supplementary Information

Supplementary Tables (1-9)

Supplementary Figures (1-11)

Supplementary Table 1 Results of multiple comparisons for α and β diversity among different regions along the latitudinal gradient. Data are presented as mean. Letters indicate different regions that differ significantly at $P < 0.05$ (n = 60 independent samples).

Diversity	Latitude	Multiple comparisons (Tukey HSD test, $P < 0.05$)						
		a	b	c	d	e	f	g
α diversity	39° N	2940.35						
	36° N	2934.35						
	31° N	2869.7	2869.7					
	42° N	2824.65	2824.65					
	45° N		2722.25					
	29° N			2527.25				
	47° N				2342.23			
	33° N				2305.05			
	53° N					1948.4		
	18° N						1489.15	
	23° N							1297.75
β diversity	33°N	0.398						
	31°N	0.395						
	29°N		0.348					
	47°N		0.342	0.342				
	18°N		0.339	0.339				
	53°N		0.331	0.331				
	36°N			0.306	0.306			
	45°N				0.291	0.291		
	42°N				0.287	0.287		
	23°N				0.272	0.272		
	39°N					0.25998		

Supplementary Table 2 One-sample two-sided *t*-test was used to compare mean β deviation against zero for 60 samples in each region after controlling for regional species pool.

Regions	18° N	23° N	29° N	31° N	33° N	36° N	39° N	42° N	45° N	47° N	53° N
Mean β deviation	124.92	62.45	128.3	148.6	133.53	70.83	63.31	74.06	73.12	115.57	162.09
P values	< 0.001	< 0.001	< 0.001	< 0.001	< 0.001	< 0.001	< 0.001	< 0.001	< 0.001	< 0.001	< 0.001

Supplementary Table 3 The proportions of significant observed β diversity compared to expected β diversity (including the proportions of observed β diversity significantly lower than expected β diversity, significantly greater than expected β diversity and no different from expected β diversity) in each region ($P < 0.05$, $n = 60$ independent samples) after controlling for γ diversity.

Regions	18° N	23° N	29° N	31° N	33° N	36° N	39° N	42° N	45° N	47° N	53° N
Significantly lower than expected β diversity	28.3%	46.7%	0	0	23.3%	51.7%	58.3%	56.7%	51.7%	20%	6.7%
Significantly greater than expected β diversity	60%	30%	98.3%	96.7%	75%	45%	36.7%	36.7%	41.7%	76.7%	86.7%
No significantly different from expected β diversity	11.7%	23.3%	1.7%	3.3%	1.7%	3.3%	5%	6.6%	6.6%	3.3%	6.6%

Supplementary Table 4 One-sample two-sided *t*-test was used to compare mean β deviation against zero for 60 samples in each region after controlling for γ diversity.

Regions	18° N	23° N	29° N	31° N	33° N	36° N	39° N	42° N	45° N	47° N	53° N
Mean β deviation	26.85	-6.07	46.57	48.2	36.19	-15.32	-20.56	-19.72	-19.79	31.09	48.4
P values	< 0.001	0.043	< 0.001	< 0.001	< 0.001	0.018	< 0.001	< 0.001	0.003	< 0.001	< 0.001

Supplementary Table 5 The proportions of significant observed β diversity compared to expected β diversity (including the proportions of observed β diversity significantly lower than expected β diversity, significantly greater than expected β diversity and no different from expected β diversity) in each region ($P < 0.05$, $n = 60$ independent samples) after controlling for regional species pool.

Regions	18° N	23° N	29° N	31° N	33° N	36° N	39° N	42° N	45° N	47° N	53° N
Significantly lower than expected β diversity	3.3%	0	0	0	0	0	1.7%	0	0	0	0
Significantly greater than expected β diversity	96.7%	100%	100%	100%	100%	100%	96.6%	100%	100%	100%	100%
No significantly different from expected β diversity	0	0	0	0	0	0	1.7%	0	0	0	0

Supplementary Table 6 Significance of environmental and spatial variables used in stepwise regression analysis across all the regions. Numbers indicate *P*-values of significant variables retained after forward-selection stepwise regression. *P*-values are evaluated using two-sided *t*-tests.

Explanatory Variable	Different regions along the latitudinal gradient (N)										
	18°	23°	29°	31°	33°	36°	39°	42°	45°	47°	53°
**Environment: Soil**											
Soil moisture				0.03							
Soil pH											
Soil organic carbon	< 0.001	0.002	< 0.001	< 0.001	0.001	0.011				0.027	0.002
Total nitrogen	0.018			0.02	0.004	0.028	0.005	0.001	< 0.001	0.002	< 0.001
Available nitrogen		0.014	0.003						0.003	0.011	
**Environment: Topography**											
Slope											
**Space**											
Latitude	0.019			0.002							0.003
Longitude			0.002		0.035	0.003	0.005	0.007		0.007	< 0,001
PCNM eigenfunctions	0.006	0.001	0.003	0.014	0.026	0.003	0.02	< 0.001	0.04	0.001	0.007

Supplementary Table 7 Summary of environmental factors at different regions along the latitudinal gradient. Mean values and coefficients of variations (CVs, %) calculated from 11 regions are shown for the following parameters: soil moisture (SM), soil organic carbon (SOC), total nitrogen (TN), available nitrogen (AN), soil pH and Slope.

Locations		SM (%)	SOC (g kg^{-1})	TN (g kg^{-1})	AN (mg kg^{-1})	soil pH	Slope (°)
18°	mean	30.94	35.43	1.97	146.96	5.05	20.56
	CV	19.98	40.83	40.35	52.96	7.68	12.3
23°	mean	34.1	41.8	1.85	104.79	3.98	20.8
	CV	19.07	39.97	56.36	50.25	8.08	15.44
29°	mean	34.82	91.18	4.93	355.68	4.88	26.01
	CV	17.52	48.74	47.17	43.04	11.82	12.79
31°	mean	35.05	74.39	4.66	573.71	6.12	25.85
	CV	16.62	38.14	49.33	61.61	9.33	19.76
33°	mean	29.07	47.14	2.91	317.34	4.91	27.93
	CV	17.95	40.79	48.15	44.09	5.6	15.92
36°	mean	23.73	41.42	2.71	261.85	6.28	24.8
	CV	21.33	26.48	22.36	21.65	3.6	12.06
39°	mean	29.36	112.91	3.16	188.3	6.72	25.1
	CV	16.27	16.66	19.28	30.24	3.27	12.13
42°	mean	36.06	95.55	7.01	502.35	5.29	7.08
	CV	13.05	29.63	23.49	28.55	8.53	12.81
45°	mean	41.39	67.18	5.84	439.09	5.69	17.48
	CV	14.97	19.53	29.72	20.61	5.28	11.82
47°	mean	43.7	120.59	6.82	412.1	5.81	14.85
	CV	20.34	37.41	48.41	41.33	15.09	13.87
53°	mean	34.7	55.67	1.86	178.82	5.43	6.44
	CV	14.5	34.45	54.39	47.99	9.1	12.72

Supplementary Table 8 Spatial autocorrelation for mean annual temperature (MAT), mean annual precipitation (MAP), mean temperature of warmest quarter (MTWQ), mean temperature of coldest quarter (MTCQ), soil moisture (SM), soil organic carbon (SOC), total nitrogen (TN), available nitrogen (AN), soil pH, elevation and slope.

Region		MAT	MAP	MTWQ	MTCQ	SM	SOC	TN	AN	soil pH	Elevation	Slope
18°	Moran's *I*	0.76	0.69	0.75	0.77	−0.08	0.009	−0.09	0.1	−0.12	0.78	0.1
	P	< 0.001	< 0.001	< 0.001	< 0.001	0.81	0.36	0.88	0.09	0.9	< 0.001	0.08
23°	Moran's *I*	0.81	0.82	0.77	0.8	0.07	−0.01	−0.02	−0.01	0.86	0.48	0.09
	P	< 0.001	< 0.001	< 0.001	< 0.001	0.09	0.48	0.53	0.47	−0.09	< 0.001	0.12
29°	Moran's *I*	0.78	0.78	0.73	0.77	−0.17	0.1	−0.09	−0.04	−0.05	0.62	0.01
	P	< 0.001	< 0.001	< 0.001	< 0.001	0.98	0.89	0.85	0.63	0.71	< 0.001	0.31
31°	Moran's *I*	0.74	0.89	0.8	0.83	−0.08	0.06	−0.04	0.05	0.09	0.73	0.02
	P	< 0.001	< 0.001	< 0.001	< 0.001	0.81	0.13	0.65	0.19	0.1	< 0.001	0.32
33°	Moran's *I*	0.67	0.74	0.68	0.68	0.01	−0.16	−0.03	−0.09	−0.11	0.84	0.04
	P	< 0.001	< 0.001	< 0.001	< 0.001	0.32	0.98	0.6	0.85	0.9	< 0.001	0.2
36°	Moran's *I*	0.83	0.72	0.84	0.75	0.01	−0.03	−0.09	−0.02	−0.12	0.15	0.09
	P	< 0.001	< 0.001	< 0.001	< 0.001	0.33	0.59	0.84	0.53	0.92	0.002	0.56
39°	Moran's *I*	0.72	0.73	0.65	0.7	0.006	−0.1	0.04	−0.04	0.07	0.7	0.08
	P	< 0.001	< 0.001	< 0.001	< 0.001	0.37	0.89	0.21	0.66	0.1	< 0.001	0.09
42°	Moran's *I*	0.79	0.78	0.8	0.76	0.006	−0.06	−0.007	0.08	−0.1	0.83	0.07
	P	< 0.001	< 0.001	< 0.001	< 0.001	0.37	0.75	0.45	0.11	0.89	< 0.001	0.32
45°	Moran's *I*	0.68	0.76	0.7	0.66	−0.02	0.02	0.09	−0.12	−0.04	0.01	0.01
	P	< 0.001	< 0.001	< 0.001	< 0.001	0.51	0.32	0.06	0.09	0.67	0.33	0.06
47°	Moran's *I*	0.7	0.76	0.62	0.68	0.09	−0.05	−0.07	−0.01	−0.08	0.64	−0.1
	P	< 0.001	< 0.001	< 0.001	< 0.001	0.08	0.69	0.78	0.47	0.82	< 0.001	0.88
53°	Moran's *I*	0.14	0.09	0.13	0.12	−0.01	−0.14	−0.11	−0.06	−0.03	0.67	0.07
	P	0.003	0.03	0.003	0.006	0.47	0.96	0.89	0.72	0.6	< 0.001	0.35

Supplementary Table 9 Significance of environmental and spatial variables used in stepwise regression analysis across all the regions. Check marks indicate explanatory variables that were included in the stepwise regression after excluding variables that were highly correlated with other variables (r > 0.80).

Explanatory variable	Different regions along the latitudinal gradient (N)										
	18°	23°	29°	31°	33°	36°	39°	42°	45°	47°	53°
**Environment: Soil**											
Soil moisture	√	√	√	√	√	√	√	√	√	√	√
Soil pH	√	√	√	√	√	√	√	√	√	√	√
Soil organic carbon	√	√	√	√	√	√	√	√	√	√	√
Total nitrogen	√	√	√	√	√	√	√	√	√	√	√
Available nitrogen	√	√	√	√	√	√	√	√	√	√	√
**Environment: Topography**											
Slope	√	√	√	√	√	√	√	√	√	√	√
**Space**											
Latitude	√			√	√	√		√	√	√	√
Longitude	√	√	√	√	√	√	√	√	√	√	√
PCNM eigenfunctions	√	√	√	√	√	√	√	√	√	√	√

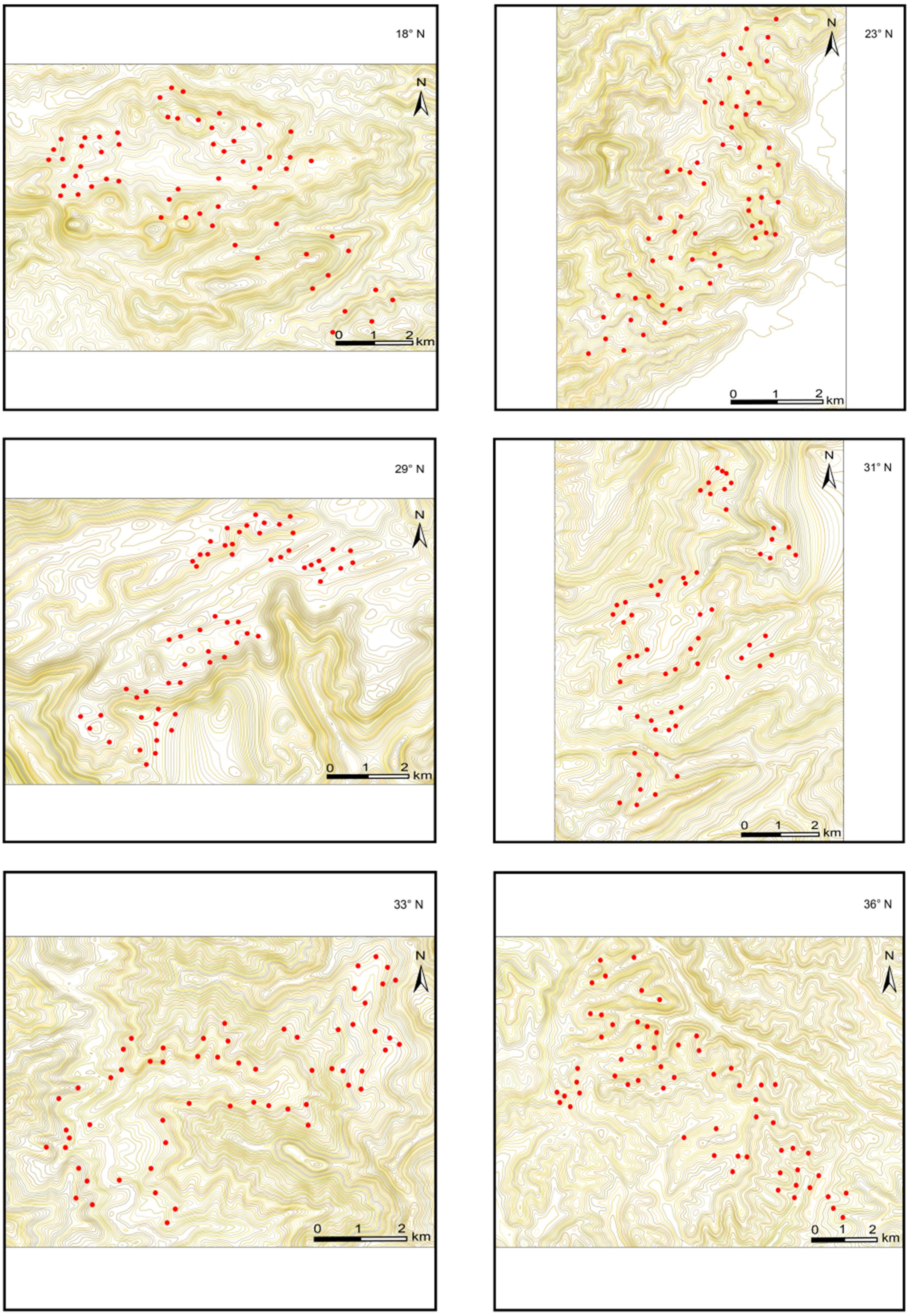
18° N
23° N
29° N
31° N
33° N
36° N
N
0 1 2 km

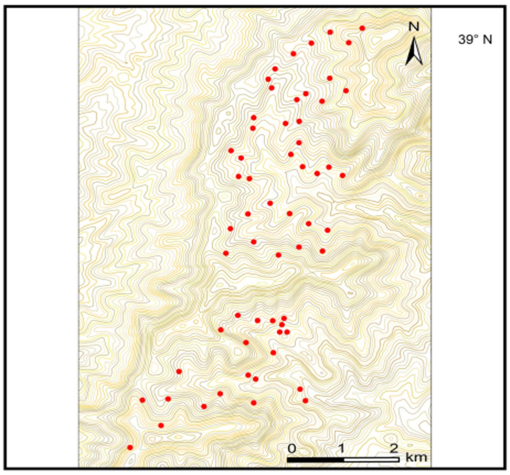
39° N
N
0 1 2 km

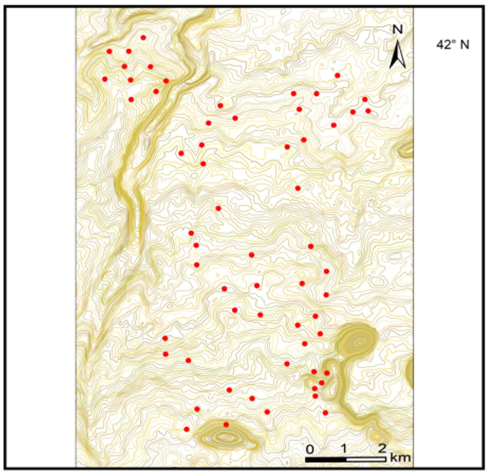
42° N
N
0 1 2 km

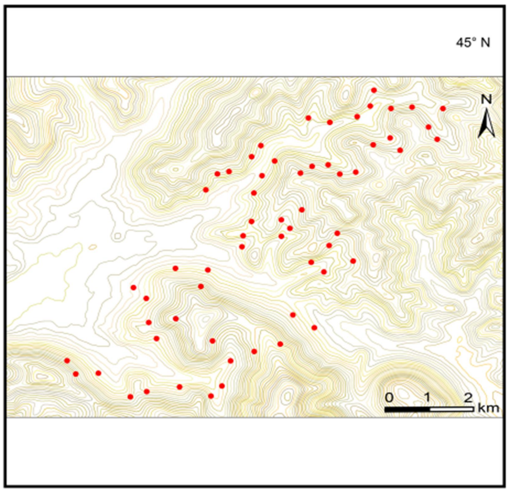
45° N
N
0 1 2 km

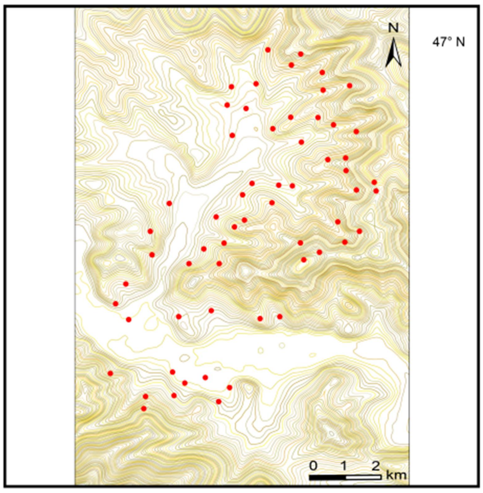
47° N
N
0 1 2 km

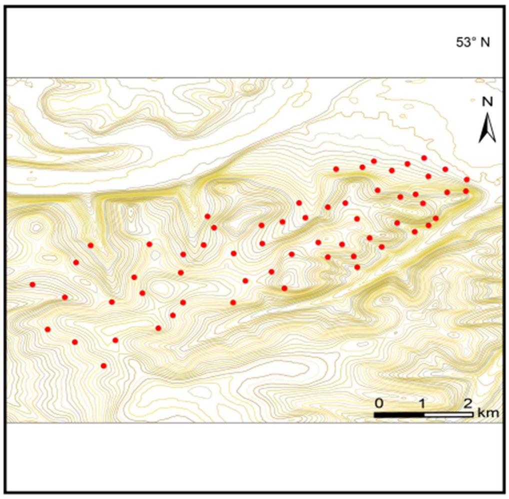
53° N
N
0 1 2 km

Supplementary Figure 1 Geographical regions and spatial distribution of sampling plots. Red points represent each 20×20 m plots in regions along the latitudinal gradient. The source data are provided as a Source Data file.

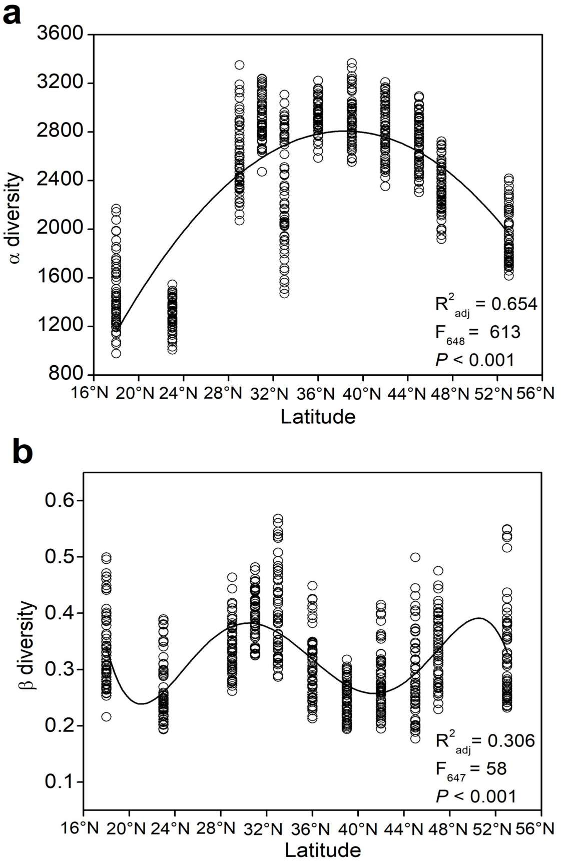

Supplementary Figure 2 Soil bacterial diversity patterns along the latitudinal gradient (excluding the outliers identified in Supplementary Figure 3)**. a** α diversity and **b** β diversity across the 11 regions along the latitudinal gradient. The source data are provided as a Source Data file. The relations between (α and β) diversity and latitude were evaluated using linear and polynomial regressions and the best polynomial fit was determined on the basis of Akaike information criterion (AIC). The *P*-values by two-sided F-test are indicated in **a** and **b** (n = 60 independent samples).

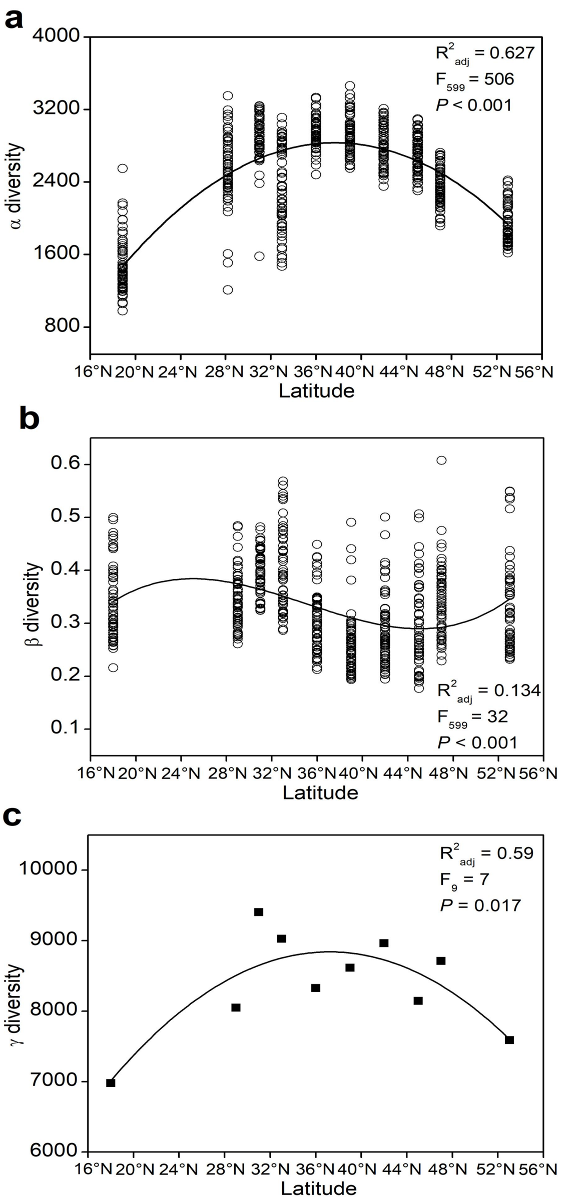

Supplementary Figure 3 Soil bacterial diversity patterns along latitude (excluding the outlier at 23°N region). a bacterial α diversity, b β diversity and c γ diversity exhibit contrasting patterns across the 10 regions along the latitudinal gradient. The relations between (α, β and γ) diversity and latitude were evaluated using linear and polynomial regressions and the best polynomial fit was determined on the basis of Akaike information criterion (AIC) and the corrected Akaike information criterion AIC (AICc) (see Methods 'Statistical analyses'). The *P*-values by two-sided F-test were indicated in **a, b** and **c** (*n* = 60 independent samples). The source data are provided as a Source Data file.

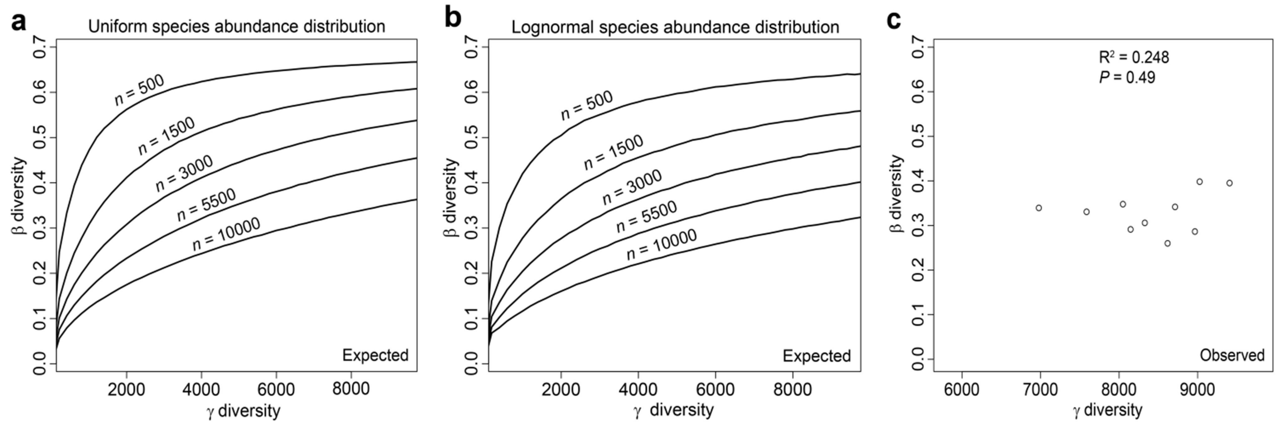

Supplementary Figure 4 The relation between β and γ diversity. **a** The expected relation between β and γ diversity produced by a simple sampling simulation using a uniform species abundance distribution. Curves represent β diversity values and '*n*' represents the number of individuals in each plot. **b** Same as **a**, but with a lognormal species abundance distribution. **c** The relation between observed β and γ diversity in empirical data from soil bacterial communities (excluding the outlier at 23°N region) along the latitudinal gradient. The *P*-values by two-sided spearman correlation are indicated in Fig **c**. The source data are provided as a Source Data file.

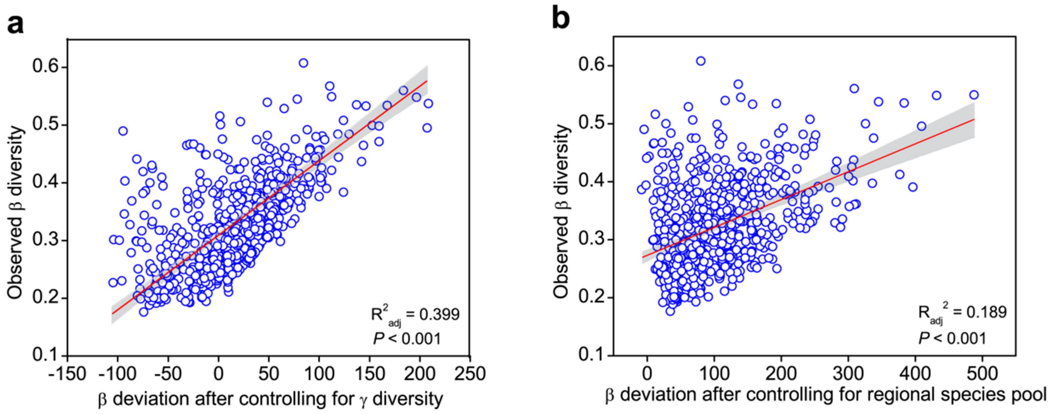

Supplementary Figure 5 The relation between observed β diversity and β deviation along the latitudinal gradient. **a** after controlling for variations in γ diversity. **b** after controlling for variations in regional species pool. The red lines indicate the estimated fit of linear regression and shaded areas show 95% confidence interval of the fit. The *P*-values by two-sided F-test were indicated in **a** and **b**. The source data are provided as a Source Data file.

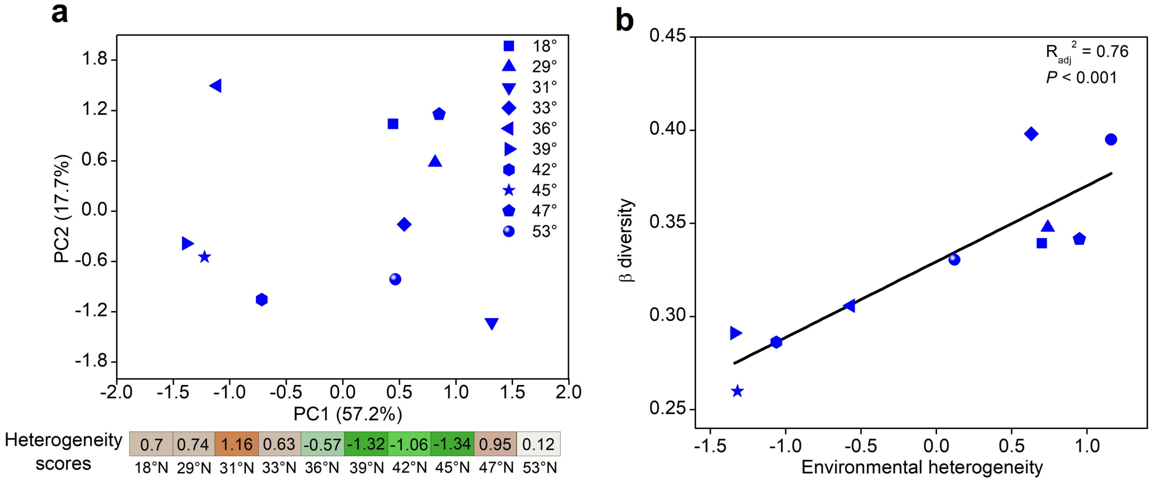

Supplementary Figure 6 The relation between β diversity and overall environmental heterogeneity. **a** The variations in total environmental heterogeneity across the 10 regions (excluding the outlier at 23°N region) along the latitudinal gradient. Principal component analysis of total environmental heterogeneity based on the coefficients of variation (CVs) of 6 environmental parameters: soil moisture, soil pH, soil organic carbon, total nitrogen, available nitrogen and slope. The score reflects the total environmental heterogeneity in each region, and a high score (orange color) represents a strong heterogeneity; a low score (green color) represents a weak heterogeneity. The score of each region is a comprehensive number of the weighted PC1 scores, PC2 scores and PC3 scores. **b** The relationship between β diversity and environmental heterogeneity across the 10 regions The relation between β diversity and overall environmental heterogeneity (excluding the outlier at 23°N region) along the latitudinal gradient was evaluated using linear regressions and the *P*-value by two-sided F-test in **b**. The source data are provided as a Source Data file.

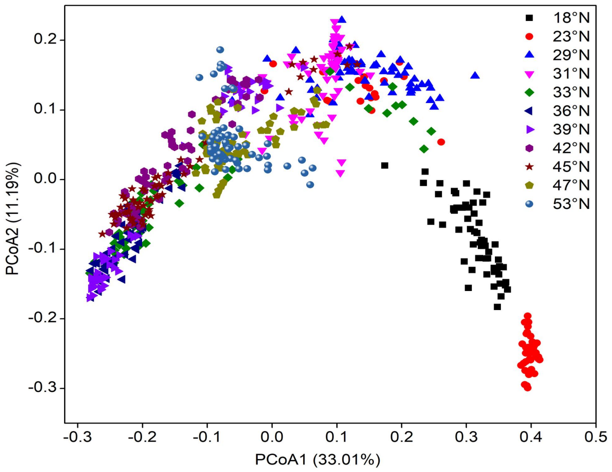

Supplementary Figure 7 Overall pattern of soil bacterial community composition along the latitudinal gradient. The source data are provided as a Source Data file.

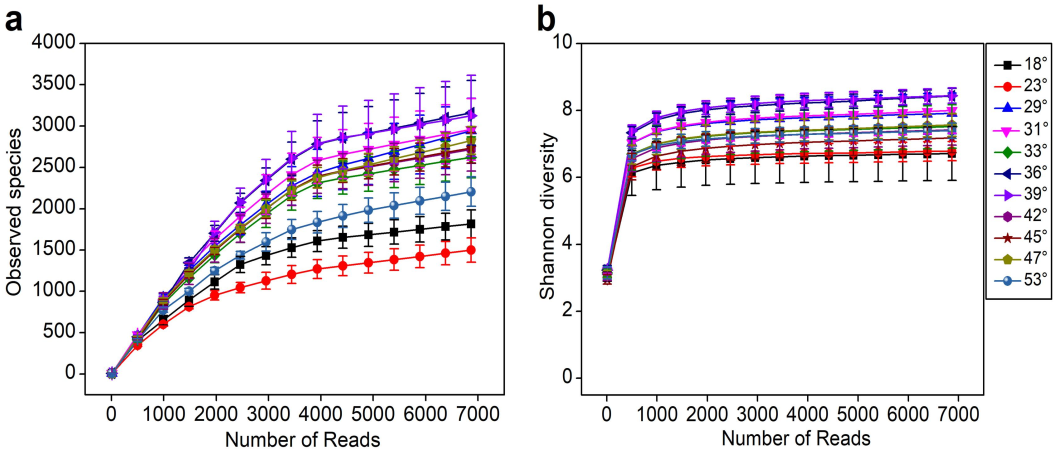

Supplementary Figure 8 Rarefaction curves for diversity. **a** Observed species and **b** Shannnon diversity. Each line represents an observed diversity in each region. Points represent means and error bars around each point indicate the standard deviation (means ± SD; n = 60 independent samples). The source data are provided as a Source Data file.

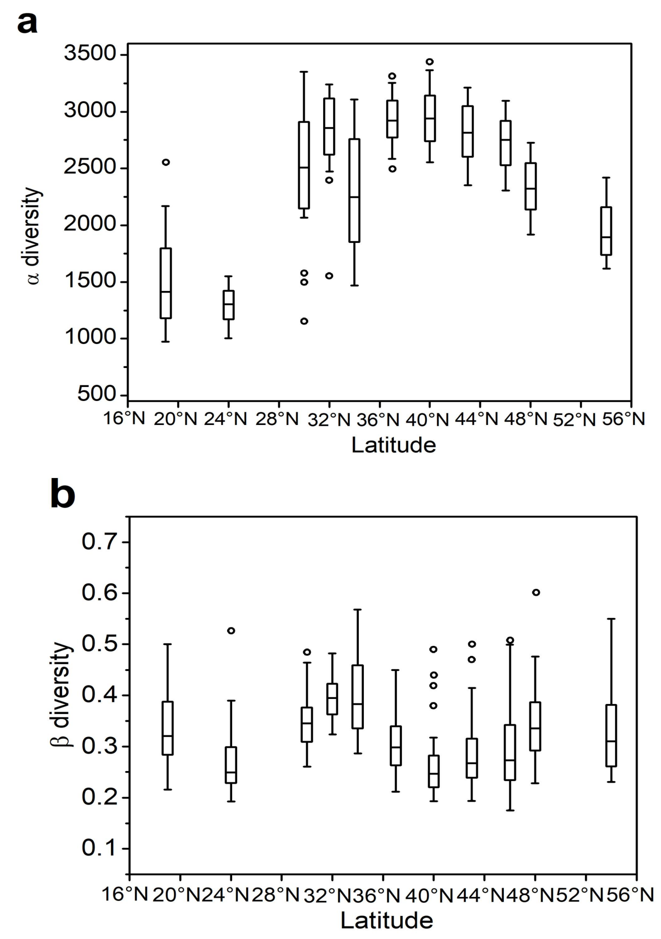

Supplementary Figure 9 The detection of potential outliers in the distribution of diversity. **a** α diversity and **b** β diversity within each region using raw data. A circle represents a potential outlier. There were 60 α diversity values in each region and the numbers of potential outliers identified in the regions of 18°, 29°, 31°, 36° and 39° were 1, 3, 2, 2 and 1, respectively. There were 60 β diversity values in each region and the numbers of potential outliers identified in the regions of 23°, 29°, 39°, 42°, 45° and 47° N were 1, 2, 4, 2, 1 and 1, respectively. The bottom and top of each box represents the first and third quartiles, and the line inside is the median. The whiskers correspond to 1.5 times the interquartile range, while data beyond the whiskers are outlying points that are plotted individually. The source data are provided as a Source Data file.

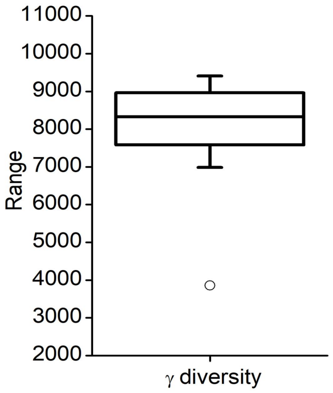

Supplementary Figure 10 The detection of potential outliers in the distribution of γ diversity. A circle represents a potential outlier that was identified at 23° N region. The bottom and top of each box represents the first and third quartiles, and the line inside is the median. The whiskers correspond to 1.5 times the interquartile range, while data beyond the whiskers are outlying points that are plotted individually. The source data are provided as a Source Data file.

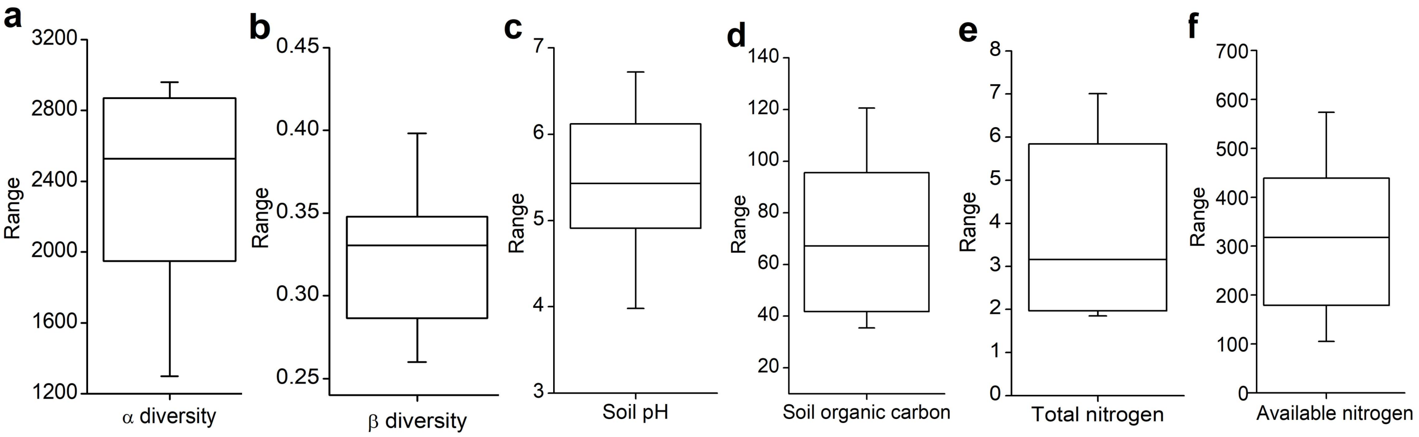

Supplementary Figure 11 The detection of potential outliers in the distribution of mean soil parameter. **a** α diversity, **b** β diversity, **c** soil pH, **d** soil organic carbon, **e** total nitrogen and **f** available nitrogen across 11 regions (an average was calculated for each region). The bottom and top of each box represents the first and third quartiles, and the line inside is the median. The whiskers correspond to 1.5 times the interquartile range. The source data are provided as a Source Data file.
