# Supplementary material for: Local community assembly mechanisms shape soil bacterial β diversity patterns along a latitudinal gradient
Source: Nat Commun. 2020 Oct 27;11:5428. doi: 10.1038/s41467-020-19228-4 (PMC7591474; doi:10.1038/s41467-020-19228-4)
Supplement: Supplementary file 7 — Reporting Summary [file 41467_2020_19228_MOESM7_ESM.pdf]

nature research

Corresponding author(s): Shirong Liu
Last updated by author(s): Sep 19, 2020

Reporting Summary

Nature Research wishes to improve the reproducibility of the work that we publish. This form provides structure for consistency and transparency in reporting. For further information on Nature Research policies, see Authors & Referees and the Editorial Policy Checklist.

Statistics

For all statistical analyses, confirm that the following items are present in the figure legend, table legend, main text, or Methods section.

n/a	Confirmed	
☐	☒	The exact sample size (*n*) for each experimental group/condition, given as a discrete number and unit of measurement
☐	☒	A statement on whether measurements were taken from distinct samples or whether the same sample was measured repeatedly
☐	☒	The statistical test(s) used AND whether they are one- or two-sided<br>*Only common tests should be described solely by name; describe more complex techniques in the Methods section.*
☒	☐	A description of all covariates tested
☐	☒	A description of any assumptions or corrections, such as tests of normality and adjustment for multiple comparisons
☐	☒	A full description of the statistical parameters including central tendency (e.g. means) or other basic estimates (e.g. regression coefficient) AND variation (e.g. standard deviation) or associated estimates of uncertainty (e.g. confidence intervals)
☐	☒	For null hypothesis testing, the test statistic (e.g. *F*, *t*, *r*) with confidence intervals, effect sizes, degrees of freedom and *P* value noted<br>*Give P values as exact values whenever suitable.*
☒	☐	For Bayesian analysis, information on the choice of priors and Markov chain Monte Carlo settings
☒	☐	For hierarchical and complex designs, identification of the appropriate level for tests and full reporting of outcomes
☐	☒	Estimates of effect sizes (e.g. Cohen's *d*, Pearson's *r*), indicating how they were calculated

Our web collection on statistics for biologists contains articles on many of the points above.

Software and code

Policy information about availability of computer code

Data collection	Soil bacterial communities were sequenced using Illumina Miseq system with Reagent Kit v2 2 × 250 bp.
Data analysis	The raw sequences were sorted based on unique sample tags and then were trimmed using QIIME Pipeline (http://qiime.org/tutorials/tutorial.html). Analyses were performed in R project 3.6.1 and The R code supporting the findings presented here is available at https://github.com/YTHHN/R-code.

For manuscripts utilizing custom algorithms or software that are central to the research but not yet described in published literature, software must be made available to editors/reviewers. We strongly encourage code deposition in a community repository (e.g. GitHub). See the Nature Research guidelines for submitting code & software for further information.

Data

Policy information about availability of data

All manuscripts must include a data availability statement. This statement should provide the following information, where applicable:

- Accession codes, unique identifiers, or web links for publicly available datasets
- A list of figures that have associated raw data
- A description of any restrictions on data availability

The datasets analysed during the current study are available in the [NCBI Sequence Read Archive by accession no.PRJNA552986] repository, [http://www.ncbi.nlm.nih.gov/bioproject/552986]. Source data for Figs. 1, 3~7 and Supplementary Figs. 1~11 can be found in the source data file. Climate attributes were obtained from the WorldClim database (www.worldclim.org).

Field-specific reporting

Please select the one below that is the best fit for your research. If you are not sure, read the appropriate sections before making your selection.

☐ Life sciences ☐ Behavioural & social sciences ☒ Ecological, evolutionary & environmental sciences

For a reference copy of the document with all sections, see nature.com/documents/nr-reporting-summary-flat.pdf

Ecological, evolutionary & environmental sciences study design

All studies must disclose on these points even when the disclosure is negative.

Study description	The study was conducted in natural forest communities along the North-South Transact of eastern China, which spans 3,700 km from north to south with latitude ranging from 53º27′N to 18º43′N and longitude ranging from 108º53′E to 122º20′E. Soil bacterial communities was investigated in 660 plots from 11 regions (60 plots at each region along the latitudinal gradient in North-South Transect of eastern China.
Research sample	Our research samples are soil bacterial communities.
Sampling strategy	We had 60 replicates per region. We based this on previous studies (Fierer, N., Jackson, R.B. 2006. The diversity and biogeography of soil bacterial communities. Proc. Natl Acad. Sci. 103, 626-631. Wang, X.B. et al. 2017. Habitat-specific patterns and drivers of bacterial β-diversity in China's drylands. ISME J. 11, 1345. Myers, J.A. et al. 2013 Beta-diversity in temperate and tropical forests reflects dissimilar mechanisms of community assembly. Ecol. Lett. 16, 151-157).
Data collection	Soil bacterial communities was investigated in 660 plots from 11 regions (60 plots at each region along the latitudinal gradient in North-South Transect of eastern China. Data collection was performed by Xiao Zhang.
Timing and spatial scale	Sampling occurred during June and July (summer) in 2015, from the south to the north along the latitudinal gradient in eastern China. We ensure all the samples are completed in summer.<br><br>Soil samples were collected from all 660 plots using a uniform sampling protocol. Each sample was a composite of six individual soil cores (2.5 cm diameter × 10 cm depth) randomly collected from the horizon within each plot.
Data exclusions	No data were excluded.
Reproducibility	Sequencing methods taken in this study are widely adopted.
Randomization	In each region, 60 plots were randomly selected. In each region, 60 plots were established randomly in a typical forest without anthropogenic or natural disturbance.
Blinding	Blinding was not relevant to this study because it did not involve a treatment group.

Did the study involve field work? ☒ Yes ☐ No

Field work, collection and transport

Field conditions	The mean annual temperature (MAT) of the transect ranges from −4.4 to 20.9°C. The mean annual precipitation (MAP) is approximatelyabout 482~ 2,449 mm.
Location	The study was conducted in natural forest communities along the North-South Transact of eastern China, which spans 3,700 km from north to south with latitude ranging from 53º27′N to 18º43′N and longitude ranging from 108º53′E to 122º20′E.
Access and import/export	All the field work campaigns were carried out in compliance with local, national and international laws.
Disturbance	Sampling plots were established randomly in a typical forest without anthropogenic or natural disturbance.

Reporting for specific materials, systems and methods

We require information from authors about some types of materials, experimental systems and methods used in many studies. Here, indicate whether each material, system or method listed is relevant to your study. If you are not sure if a list item applies to your research, read the appropriate section before selecting a response.

Materials & experimental systems

n/a	Involved in the study
☒	☐ Antibodies
☒	☐ Eukaryotic cell lines
☒	☐ Palaeontology
☒	☐ Animals and other organisms
☒	☐ Human research participants
☒	☐ Clinical data

Methods

n/a	Involved in the study
☒	☐ ChIP-seq
☒	☐ Flow cytometry
☒	☐ MRI-based neuroimaging
